# Effect of Daily Iron Supplementation on Infantile Iron Homeostasis in Preterm Infants

**DOI:** 10.3389/fped.2021.687119

**Published:** 2021-05-28

**Authors:** Mingyan Li, Ying Lv, Jionghuan Ying, Lin Xu, Weijun Chen, Quan Zheng, Chai Ji, Jie Shao

**Affiliations:** ^1^Department of Pediatric Health Care, The Children's Hospital, Zhejiang University School of Medicine, National Clinical Research Center for Child Health, Hangzhou, China; ^2^Department of Pediatrics, Cixi People's Hospital, Cixi, China

**Keywords:** iron deficiency, iron deficiency anemia, preterm infants, iron supplementation, correlative factors, growth

## Abstract

**Objective:** The aim of this study was to investigate the effects of unified iron supplementation and identify the factors related to the iron homeostasis among preterm infants.

**Method:** A total of 250 preterm infants were divided into neonatal anemic (NA, *n* = 154) and non-neonatal anemic group (NNA, *n* = 96). Iron supplements at a dose of 2 mg/kg per day were given from 40 weeks' gestational age to 6 months. Iron status parameters were measured at 3 and 6 months, respectively. Prevalence of iron deficiency (ID) and iron deficiency anemia (IDA), and the correlated factors were analyzed. Growth and side-effects were monitored.

**Results:** There were no significant differences for the prevalence of ID or IDA between the two groups. Multivariate regression analyses showed that higher Hb at birth and early treatment of blood transfusion reduced the risk of ID/IDA at 3 months (all *p* < 0.05); while higher level of Hb at 3 months (*p* = 0.004) and formula feeding reduced the occurrence of ID/IDA at 6 months (*p* < 0.05); males had a 3.35 times higher risk to develop ID/IDA than girls (*p* = 0.021). No differences in growth and side effects were found.

**Conclusion:** A daily dose of 2 mg/kg iron supplement is beneficial to maintain iron homeostasis in majority preterm infants within 6 months regardless of their neonatal anemia history. Under the routine iron supplementation, Hb level at birth and at 3 months, early treatment of blood transfusion, gender and feeding patterns are the major factors affecting the prevalence of ID/IDA among preterm infants in infancy.

## Introduction

Iron is an essential micronutrient for myriad of biochemical and developmental processes including those involving neurodevelopment. Iron deficiency (ID) is the most prevalent single nutrient deficiency, which can be easily diagnosed and treated. In spite of this, about 800 million women and children worldwide had anemia, nearly half of whom were caused by ID (1). Numerous studies demonstrated that ID not only causes anemia, but more importantly, adversely affects neurodevelopment in rats, primates and humans due to its essential role in myelination, dendritic growth, synaptogenesis, neurotransmission and monoamine metabolism (2–4). These pathophysiological changes will ultimately result in short and long term multiple domains of neurologizing abnormalities, including poorer motor, cognitive and learning abilities, as well as social emotional functioning (5–7). Thus, ID is considered to be an important public health problem in both developed and developing countries.

The prevalence of ID peaks in infancy. Infants, especially preterm infants, are at an elevated risk of ID/IDA for their low endowment of iron stores at birth, early onset of erythropoiesis, rapid catch-up growth, iatrogenic blood loss and limited dietary sources of iron (8). There is an evidence that preterm infants with a birth weight (BW) <1,830 g/L and a serum ferritin (SF) <155 ug/L in the 1st week of life had a 26.4 times higher risk of developing ID (9). Due to the critical role of iron in growth and central nervous system development, and the increased risk of ID/IDA in preterm infants, specific iron supplementation is of great importance for this extremely vulnerable population.

As we know, human brain develops rapidly in the last trimester, during which time the amount of iron absorbed in the central nerves system also reaches its peak. When the body is in a state of negative iron balance, iron reserves in the brain will be reduced and preferentially supplied for erythropoiesis. Although the subsequent iron supplementation in infancy can improve infant's iron status (10–12), the benefit on brain was limited (2, 13, 14). Therefore, it makes more sense for premature infants to maintain iron balance in early postnatal life, which equivalent to the peak period of iron-dependent brain development, than in later infancy (15).

However, in considerate of the poorly development antioxidant capacity in preterm infants and the potential risk of peroxidation damage caused by iron overload (16, 17), iron supplementation should be cautious in this population. To date, there is still a lack of global consensus recommendations on iron supplement for premature infants. Indications, doses, time of initiation and duration of iron supplement are heterogeneity in clinical practice in different countries (16, 18). In China, routine iron supplementation is recommended for preterm infants from the first 2~4 weeks after birth to the 12th month of corrected age, with a dosage of 2 mg/(kg·d) (19). But data relating to the outcomes of this preventive approach is very limited. More evidence-based medicine support is needed to further verify whether the current universal iron supplementation is appropriate for all preterm infants with different iron status during early postnatal life. In order to fill in the gaps, we conducted the present study to gain insight into the efficiency of this general supplementation on iron homeostasis in preterm infants with and without neonatal anemia, and to investigate the effects of iron supplementation on their growth in the first half year of life. We predicted that elementary iron supplementation of 2 mg/(kg·d) beginning in early postnatal life and continuing in infancy would improve the infantile iron status of preterm infants.

## Materials and Methods

### Study Design

This was a follow-up study of general iron supplementation given from 40 weeks' gestational age (GA) to 6 months of age to preterm infants with or without neonatal anemia. The intervention plan was based on the Chinese expert consensus (19) which has been commonly used in our clinical practice since 2018. The study was conducted at Children's Hospital Zhejiang University School of Medicine from May 2018 to Oct 2019. Data were obtained in a course of a study in the same area of the developmental effects of fetal-neonatal ID in premature infants. Outcomes were assessed at 3 and 6 months.

### Participants

A total of 266 preterm infants attending at the Follow-up Clinic for High-risk Infant were enrolled. The subjects had a GA of 27–36 weeks. Based on the lowest hemoglobin(Hb) recorded in the medical records during their neonatal hospitalization, infants were divided into two groups: those with hemoglobin(Hb) <145 g/L within 28 days after birth were classified as neonatal anemic group (NA), and the others were classified as non-neonatal anemic (NNA) group. Exclusion criteria were hemoglobinopathies, history of received erythropoietin, cyanotic heart disease, congenital intrauterine infection, congenital malformations, and hemolytic or metabolic diseases.

All the enrolled infants were required to have a monthly follow-up since 40 weeks' GA, and provided with iron supplements (2 mg/kg per day iron as oral iron proteinsuccynilate [Lee's Pharmaceutical Holdings Limited, Hong Kong]) from then on. Preterm infants in this study cohort were not introduced to have complementary food, so the total daily iron intake was calculated as the sum of iron supplements and the iron from formula and/or breast milk fortifier. During the follow-up visits, parents' compliance with iron supplementation was monitored by a designated nurse using a questionnaire. Poor compliance with the intervention was defined as <70% of doses.

### Data Collection

During the first interview, parents were required to complete a questionnaire covering the information regarding demographics, maternal complications during pregnancy (gestational diabetes, pregnancy induced hypertension, anemia), family history of genetic and chronic diseases, parental education, occupation and household income. Meanwhile, perinatal data, including BW, Apgar scores, GA, neonatal complications (intracranial hemorrhage, necrotizing enterocolitis, alimentary tract hemorrhage and enteritis anaphylactica et al.), as well as information about hemoglobin (Hb, including values tested at birth and the day before discharge, and the lowest value during neonatal period), history of iron supplementation and/or blood transfusion were all collected from neonatal medical records.

At each visit, information regarding the type of feeding (breast feeding with or without fortifier, regular or preterm formula feeding and mixed feeding), amount of milk intake and anthropometric data were collected. Anthropometric data were evaluated according to corrected age by using WHO references (20). Growth velocity was indicated by weight gain per day, calculated as (weight at assessment- birth weight)/chronological age. During the interview, parents were also asked to fill a checklist including the following events that occurred since the last follow-up: watery diarrhea (>5 episodes of watery stools per day), loose stools, hard stools, vomiting (>5 episodes per day), crying, rash and fatigue. Data of 3 and 6 months were used for analysis.

### Blood Collection and Measurement

Venous blood samples were collected at 3 and 6 months. Serum ferritin (SF), serum iron, total iron binding capacity (TIBC), Hb, mean cell volume (MCV), Red blood cell volume distribution width (RDW) and C-reactive protein (CRP) were measured. Approximately 3 ml of venous blood was collected in plain and EDTA containers. EDTA-treated blood was used for Hb and MCV analysis. Blood collected in plain tubes was centrifuged at 3,000 rpm for 5 min to separate serum. Separated serum was transferred to a fresh vial and was used for the estimation of SF levels.

Hb, MCV, and RDW were analyzed by auto-analyzer (Sysmex SE-9000 Auto Hematology Analyzer, Kobe, Japan). Serum iron and TIBC were measured by chemiluminescent immunoassay (Beckman Coulter Chemistry Analyzer AU5800, Japan). SF level was determined by electrochemiluminescence immunoassay (Cobas 6000-601, roche, Switzerland) and CRP was measured by rate nephelometry using a QuikRead 101 instrument (Orion Diagnostics). Transferrin saturation (TS) was calculated from iron and transferrin levels.

ID is diagnosed if two or more of the following outliers are met: MCV <74 fl, RDW >14%, ferritin <12 μg/L, serum iron <10.74 umol/L, TS <12% and TIBC > 62.65 umol/L. IDA is defined as ID plus anemia, and as Hb <90 g/L and <110 g/L at 3 months and 6 months, respectively. Iron overload is defined as SF >370 ug/L (18, 21, 22).

### Statistical Analysis

Statistical analyses were performed using SPSS Statistics 16.0. Means (SD) and proportions were calculated to describe the composition of the target subjects. Comparisons of background characteristics and iron status between groups were analyzed as follows: Categorical outcome variables were analyzed by Chi-square test with continuity correction or Fisher exact test (when cell frequencies were <5) as applicable. All registered continuous variables were checked for normality and skewness using Kolmogorov-Smimov test. Normally distributed numerical variables were compared by independent samples *t*-test after evaluating equality of variance by Levene test and those with skewed distribution were compared by Mann–Whitney *U*-test. To identify the differences in anthropometric measures between NA and NNA groups, analysis of covariance (ANCOVA) was conducted, controlling for GA and BW. Multiple logistic regression analysis was used to analyze correlative factors for ID/IDA. Statistical significance was defined as a *p* < 0.05.

## Results

### Sample Characteristics

**Figure 1:**
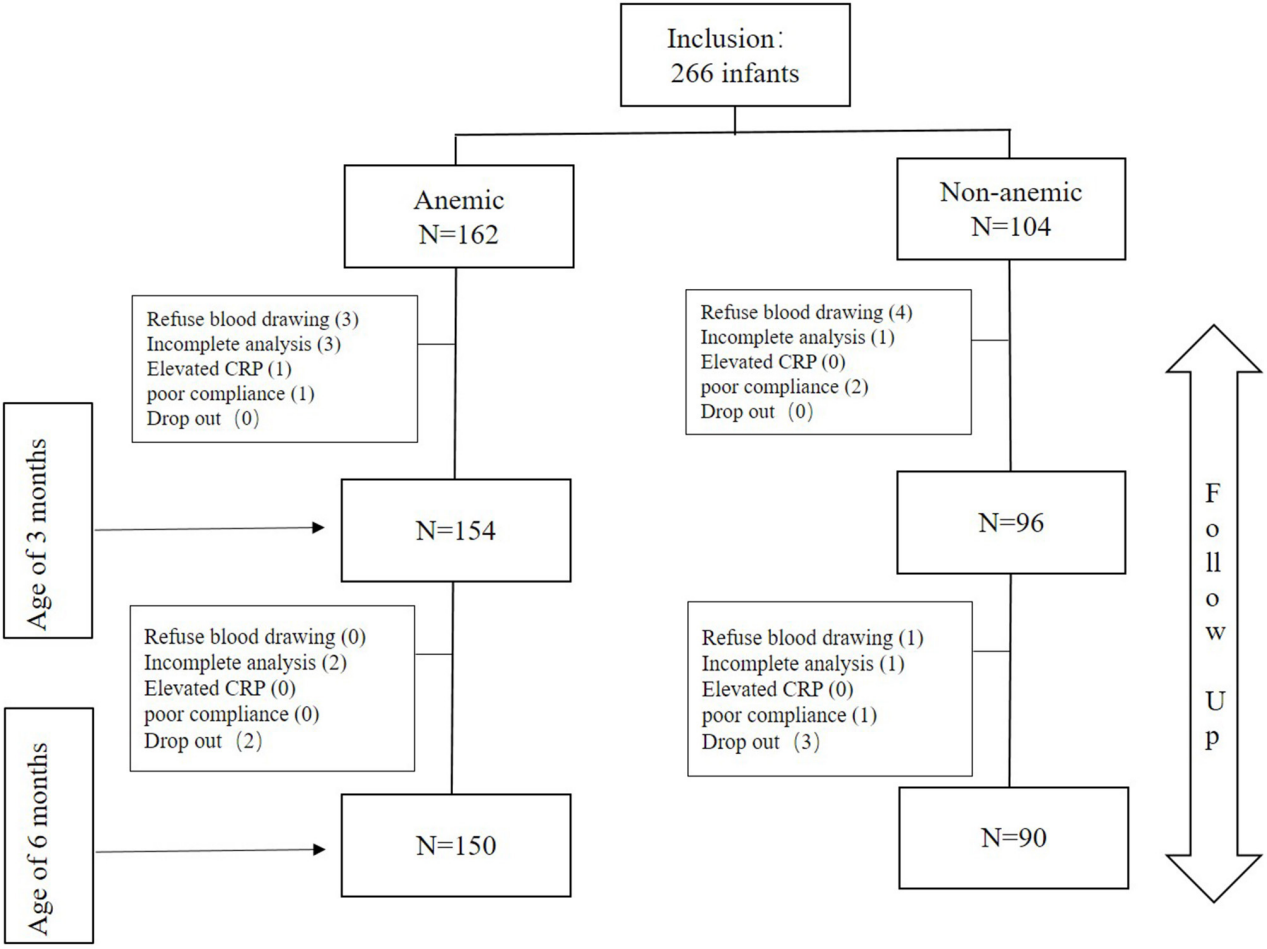
Flow chart of the study population.

In this study, a total of 266 preterm infants were enrolled, including 162 cases of NA and 104 cases of NNA. Among them, 15 dropped out of the study at 3 months for the reasons that: parents' refusal to have their children blood-drawn (*n* = 7), incomplete blood samples or sample hemolysis that could not be processed (*n* = 4), elevated CRP (*n* = 1), poor compliance (*n* = 3). At 6 months, another 10 infants dropped out due to: refusal of blood drawing (*n* = 1), incomplete blood samples (*n* = 3), poor compliance (*n* = 1), family lost contact (*n* = 5). The baseline characteristics of excluded infants were similar with those who completed the whole follow-up period (data not shown). Finally, a total of 250 3-month-old (154 in the NA group vs. 96 in the NNA group) and 240 6-month-old infants (150 in the NA group vs. 90 in the NNA group) were enrolled for data analysis (Figure 1).

The background characteristics were compared between the two groups (Table 1). The groups differing in GA, BW, and Hb values (including the lowest value obtained during neonatal period, the values tested at birth and the day before discharge). The mean values of the above parameters in the NA group were all significantly lower than those in NNA group (all *p* < 0.001).

**Table 1:** Infant and family characteristics by group.

**Group**	**NA**	**NNA**	***t*, *z*, or χ^**2**^**	***p-*value**
*N*	154	96		
**Infant characteristics**				
BW, g^a^	1618.5 ± 443.7	2152.1 ± 400.8	−9.59	<0.001
GA, wk^b^	32.0 (3.8)	34.3 (2.1)	−8.16	<0.001
Male, *n*/total (%)^c^	93 (60.4)	50 (52.1)	1.67	0.197
Cesarean section, *n*/total (%)^c^	38 (24.7)	31 (32.3)	1.72	0.190
Hb at birth (g/L)^a^	168.1 ± 31.5	182.1 ± 25.9	−3.82	<0.001
Lowest Hb during neonatal period (g/L)^b^	112.5 (28.8)	162.5 (13.0)	−12.32	<0.001
Hb before discharge (g/L)^b^	108 (30.0)	162.5 (22.5)	−11.92	<0.001
History of blood transfusion, *n*/total (%)^c^	46/154 (29.9)	0/96 (0.0)	35.14	<0.001
History of iron supplementation, *n*/total (%)^c^	85/154 (55.2)	3/96 (3.1)	70.29	<0.001
Digestive diseases, *n*/total (%)^c^	20 (13.0)	1 (1.1)	10.97	0.001
Intracranial hemorrhage, *n*/total (%)^c^	50 (32.5)	19 (19.8)	4.76	0.029
Exclusive breastfeeding at 6 mo, *n*/total (%)^c^	58 (37.7)	36 (40.0)	0.13	0.717
**Family characteristics**				
Maternal age at delivery, y^a^	30.4 ± 4.4	31.4 ± 4.1	−1.80	0.074
Complications during pregnancy, *n*/total (%)^c^	44 (28.6)	17 (17.7)	3.78	0.052
Maternal education, ≥high school, *n*/total (%)^c^	120 (77.9)	78 (81.3)	0.40	0.528
Family income ≤50,000 yuan/y, *n*/total (%)^c^	15 (9.7)	6 (6.3)	0.94	0.333

*NA, neonatal anemic; NNA, neonatal non-anemic; BW, birth weight; GA, gestational age; Hb, hemoglobin*.

a *Values are mean (SD). Statistical significance was determined by independent samples t-test*.

b *Values are median (IQR). Statistical significance was determined by Mann–Whitney U-test*.

c *Data are presented as percentages; p-values are based on Chi-square test with continuity correction or Fisher exact test with cell frequencies <5*.

In addition, due to the small number of cases, we combined infants with maternal gestational diabetes, anemia and/or hypertension into a category of infants with maternal complication; those with necrotizing enterocolitis, gastrointestinal bleeding and/or enteritis anaphylactica into a category of infants with digestive system diseases. By data analysis, we found that infants in the NA group had a higher incidence of digestive diseases (*p* = 0.001) and intracranial hemorrhage (*p* = 0.029), and also had a higher proportion receiving blood transfusion and/or oral iron supplementation during neonatal hospitalization (all *p* < 0.001). A total of 10 preterm infants (4%) had a history of multiple blood transfusions (≥2). There were no differences in other background characteristics between the two groups.

### Prevalence of ID/IDA With Daily Iron Supplementation

The iron status parameters were presented in Table 2. RDW and/or TS of the NA group were higher than those in the NNA group at 3 and 6 months (all *p* < 0.05). The remaining indicators were similar between two groups.

**Table 2:** Infant iron status at 3 and 6 months' assessments by group.

**Biochemical results**	**NA**	**NNA**	***t*, *z*, or *x*^**2**^**	***p-*value**
3 months (N)	154	96		
Age^b^	3.2 (0.5)	3.1 (0.4)	−1.11	0.268
Hb (g/L)^a^	102.3 ± 9.9	104.5 ± 10.2	−1.73	0.085
MCV(fl)^b^	85.9 (6.7)	84.6 (6.6)	−1.59	0.113
RDW (%)^b^	13.6 (1.6)	13.2 (1.6)	−2.59	0.009
SF (μg/L)^b^	41.4 (40.7)	38.9 (47.9)	−0.16	0.877
Serum iron (μmol/L)^a^	14.4 ± 4.3	13.4 ± 3.8	1.83	0.069
TS (%)^a^	25.0 ± 10.9	21.9 ± 9.3	2.35	0.020
TIBC (μmol/L)^b^	54.2 (9.9)	55.5 (10.9)	−1.34	0.181
IDA, n/total *n*(%)^c^	5/154 (3.2)	2/96 (2.1)	0.31	0.711
ID, n/total *n*(%)^c^	16/154 (10.4)	15/96 (15.6)	1.49	0.222
6 months (N)	150	90		
Age^b^	6.1 (0.4)	6.1 (0.5)	−1.32	0.187
Hb (g/L)^a^	119.8 ± 10.7	118.6 ± 9.3	0.85	0.395
MCV(fl)^b^	80.4 (5.5)	80.4 (4.7)	−0.75	0.451
RDW (%)^b^	12.9 (1.4)	13.0 (1.5)	−1.04	0.300
SF (μg/L)^b^	25.6 (18.3)	26.7 (20.4)	−0.52	0.610
Serum iron (μmol/L)^a^	15.1 ± 4.0	14.0 ± 4.4	1.94	0.054
TS (%)^b^	19.0 (8.5)	18.0 (8)	−2.02	0.044
TIBC (μmol/L)^a^	56.5 ± 8.7	55.1 ± 7.4	1.34	0.183
IDA, n/total *n*(%)^c^	12/150 (8.0)	7/90 (7.8)	0.00	0.951
ID, n/total *n*(%)^c^	10/150 (6.7)	8/90 (8.9)	0.40	0.527

*MCV, mean cell volume; RDW, red blood cell volume distribution width; SF, serum ferritin; TS, transferrin saturation; TIBC, total iron binding capacity; ID, iron deficiency; IDA, iron deficiency anemia*.

a *Values are mean (SD). Statistical significance was determined by independent samples t-test*.

b *Values are median (IQR). Statistical significance was determined by Mann–Whitney U-test*.

c *Data are presented as percentages; p-values are based on Chi-square test with continuity correction or Fisher exact test with cell frequencies <5*.

At 3 months, ID was present in 16 (10.4%) and 15 (15.6%) infants in the NA and NNA group, while IDA was present in 5 (3.2%) and 2 (2.1%) infants, respectively. The incidence of ID decreased, and the incidence of IDA increased with age increasing for both groups. At the age of 6 months, ID was present in 10 cases (6.7%) and 8 cases (8.9%), while IDA was present in 12 cases (8.0%) and 7 cases (7.8%) in the NA and NNA group, respectively. However, no statistically significant overall group differences regarding the prevalence of ID/IDA were observed at two time points (all *p* > 0.05). The prevalence of anemia at 6 months in both groups was much lower than that of the general population in the same area (20.8%) (23).

In order to understand the effects of iron supplementation and/or blood transfusion during neonatal hospitalization on the effectiveness of infantile general iron supplementation in premature infants, we divided the NA group into two subgroups: infants with a history of iron supplementation and/or blood transfusion during neonatal hospitalization (NAT) and infants without a history of iron supplementation and/or blood transfusion (NAU). As shown in Table 3, the lowest value of Hb tested during neonatal period of the NAT group was significantly lower than that of the NAU (*p* < 0.001). With daily iron supplementation, the Hb level and the prevalence of ID and IDA were similar between the two subgroups at the 3- and 6-month testing (all *p* > 0.05). Other parameters, including MCV, RDW, serum iron, TS and TIBC were all within the normal range in two groups.

**Table 3:** Comparison of iron status between NAT and NAU group at 3 and 6 months.

**Biochemical results**	**NAT**	**NAU**	***t*, *z*, or χ^**2**^**	***p-*value**
3 months (N)	102	52		
Lowest Hb during neonatal period (g/L)^a^	108.2 ± 17.2	121.5 ± 15.1	−4.73	0.000
Hb (g/L)^a^	101.8 ± 9.9	103.2 ± 9.8	−0.85	0.400
MCV(fl)^b^	86.0 (6.3)	85.0 (7.9)	−1.15	0.250
RDW (%)^b^	13.7 (1.5)	13.5 (1.8)	−0.68	0.490
SF (μg/L)^b^	39.7 (41.9)	45.4 (35.5)	−0.66	0.510
Serum iron (μmol/L)^a^	14.4 ± 3.8	13.3 ± 5.1	−0.07	0.940
TS(%)^a^	26.5 ± 10.8	22.1 ± 10.4	2.43	0.200
TIBC (μmol/L)^b^	53.7 (11.1)	54.6 (11.6)	−1.86	0.060
IDA, n/total *n*(%)^c^	3/102 (2.9%)	2/52 (3.8%)	0.09	1.000
ID, n/total *n*(%)^c^	8/102 (7.8%)	8/52 (15.3%)	2.10	0.147
6 months (N)	101	49		
Hb (g/L)^a^	120.1 ± 10.9	119.1 ± 10.3	0.52	0.600
MCV (fl)^b^	81.1 (2.1)	78.6 (4.5)	−2.97	0.003
RDW (%)^b^	12.8 (1.3)	13.1 (1.4)	−2.09	0.037
SF (μg/L)^b^	25.6 (19.0)	25.3 (18.0)	−0.06	0.950
Serum Iron (μmol/L)^a^	14.6 ± 3.9	16.1 ± 4.2	−2.19	0.030
TS(%)^b^	18.0 (8.0)	21.1 (11.5)	−2.72	0.007
TIBC(μmol/L)^a^	57.5 ± 9.6	54.5 ± 6.1	1.97	0.050
IDA, n/total *n*(%)^c^	7/101 (6.9%)	5/49 (10.2%)	0.46	0.529
ID, n/total *n*(%)^c^	6/101 (5.9%)	4/49 (8.2%)	0.25	0.729

*NAT, infants with a history of iron supplementation and/or blood transfusion during neonatal hospitalization; NAU, infants without a history of iron supplementation and/or blood transfusion during neonatal hospitalization*.

a *Values are mean (SD). Statistical significance was determined by independent samples t-test*.

b *Values are median (IQR). Statistical significance was determined by Mann–Whitney U-test*.

c *Data are presented as percentages; p values are based on Chi-square test with continuity correction or Fisher exact test with cell frequencies <5*.

### Factors Associated With ID

To identify factors associated with ID/IDA in the case of daily iron supplementation among premature infants, multivariable logistic regression analysis was performed, with GA, BW, Hb (lowest value during neonatal period, and the values tested at birth, before discharge and at 3 months), sex, maternal complications, neonatal digestive system diseases, intracranial hemorrhage, neonatal history of external iron sources and blood transfusion, growth velocity and feeding patterns as covariates.

Through analysis, we found that Hb at birth and history of blood transfusion during neonatal hospitalization were important factors influencing the prevalence of ID/IDA at 3 months (Table 4). Preterm infants with higher Hb level at birth were less likely to develop ID/IDA than those with lower Hb level (OR = 0.98, 95% CI 0.96–1.00, *p* = 0.012); and those with a history of blood transfusion were less likely to have ID/IDA in early infancy (OR = 0.03, 95% CI 0.00–0.45, *p* = 0.011).

**Table 4:** Factors relating to ID/IDA at 3 months.

**Variable**	**Category**	***p-*value**	**OR^a^**	**95% CI^b^**
GA (weeks)		0.450	0.88	(0.64, 1.22)
BW (g)		0.754	1.00	(1.00, 1.00)
Growth velocity at 3 months (g/d)		0.147	1.05	(0.98, 1.13)
Hb at birth (g/L)		0.012	0.98	(0.96,1.00)
Lowest Hb during neonatal period (g/L)		0.301	0.98	(0.94, 1.02)
Hb before discharge (g/L)		0.111	1.04	(0.99, 1.08)
Sex	Female			
	Male	0.501	1.33	(0.58, 3.02)
Mother complications	No			
	Yes	0.184	1.76	(0.77, 4.04)
Digestive system diseases	No			
	Yes	0.787	0.80	(0.15, 4.19)
Intracranial hemorrhage	No			
	Yes	0.230	1.67	(0.72, 3.85)
Iron supplementation	No			
	Yes	0.826	0.88	(0.29, 2.70)
Blood transfusion	No			
	Yes	0.011	0.03	(0.00, 0.45)
Feeding patterns at 3 months	Breast feeding			
	Intensive feeding	0.536	1.40	(0.48, 4.03)
	Formula feeding	0.177	0.33	(0.07, 1.65)
	Mixed feeding	0.070	0.40	(0.15, 1.08)

a *OR, odds ratio*;

b *CI, confidence interval. This table shows the ORs with 95% CIs for the variables that were associated with ID/IDA at the postnatal age of 3 months*.

At 6 months, the occurrence of ID/IDA was closely related to sex, 3-month's Hb and feeding patterns (Table 5). Boys had a 3.35 times higher risk to develop ID/IDA compared with girls (OR = 3.35, 95% CI 1.20–9.36, *p* = 0.021). Infants with higher Hb at 3 months were less likely to have ID/IDA at 6 months (OR = 0.94, 95% CI 0.90–0.98, *p* = 0.004). In addition, compared with exclusively breast-fed infants, infants who were fortified-fed (OR = 0.06, 95% CI 0.01–0.57, *p* = 0.015), conventional formula-fed (OR = 0.17, 95% CI 0.04–0.68, *p* = 0.012) or mixed-fed (OR = 0.18, 95% CI 0.06–0.54, *p* = 0.002) had a lower risk of ID/IDA. Other factors, including GA, BW, growth velocity, external iron sources during neonatal hospitalization, as well as mother and neonatal complications were no confounders for the observed associations.

**Table 5:** Factors associate with ID/IDA at 6 months.

**Variable**	**Category**	***p-*value**	**OR**	**95% CI**
GA (weeks)		0.100	1.36	(0.94, 1.95)
BW (g)		0.053	1.00	(1.00, 1.00)
Growth velocity at 6 months (g/d)		0.711	0.98	(0.86, 1.11)
Hb at birth (g/L)		0.067	0.98	(0.97, 1.00)
Lowest Hb during neonatal period (g/L)		0.412	1.02	(0.98, 1.05)
Hb before discharge (g/L)		0.921	1.00	(0.97, 1.03)
Hb at 3 months (g/L)		0.004	0.94	(0.90, 0.98)
Sex	Female			
	Male	0.021	3.35	(1.20, 9.36)
Mother complication	No			
	Yes	0.702	1.20	(0.47, 3.10)
Digestive system disease	No			
	Yes	0.619	1.47	(0.33, 6.61)
intracranial hemorrhage	No			
	Yes	0.454	0.66	(0.23, 1.94)
Iron supplementation	No			
	Yes	0.732	1.23	(0.38, 4.02)
Blood transfusion	No			
	Yes	0.449	0.55	(0.12, 2.56)
Feeding patterns at 6 months	Breast feeding			
	Intensive feeding	0.015	0.06	(0.01, 0.57)
	Formula feeding	0.012	0.17	(0.04, 0.68)
	Mixed feeding	0.002	0.18	(0.06, 0.54)

*This table shows the ORs with 95% CIs for the variables that were associated with ID/IDA at the postnatal age of 6 months*.

### Anthropometry at Follow-Up

The anthropometric outcomes at 3 and 6 months corrected age were compared between the two groups together with the calculated weight gain (Table 6). No significant differences were detected between NA and NNA group (all *p* > 0.05).

**Table 6:** Infant anthropometric measurements^a^ at 3 and 6 months corrected age by group.

**Group**	**NA**	**NNA**	***F***	***p-*value**
**3 months**				
Weight (kg)	6.4 (0.8)	6.6 (0.8)	0.10	0.754
length (cm)	60.4 (2.2)	60.9 (2.6)	0.14	0.713
Head circumference (cm)	40.0 (1.4)	40.5 (1.4)	0.33	0.569
Weight gain (g/d)	31.5 (4.3)	33.7 (5.1)	0.17	0.684
**6 months**				
Weight (kg)	7.9 (0.9)	8.1 (1.0)	0.55	0.458
Length (cm)	66.8 (2.1)	66.9 (2.4)	0.36	0.547
Head circumference (cm)	42.9 (1.5)	43.2 (1.6)	0.18	0.674
Weight gain (g/d)	16.6 (6.0)	16.5 (4.6)	0.90	0.345
Weight gain (g/d)	25.9 (3.4)	26.5 (3.8)	0.39	0.535

a *Values are shown as means ± SD, Statistical significance was determined by analysis of covariance with GA and BW as covariates*.

### Supplement Tolerance and Adverse Events

In our study, blood tests at 3 and 6 months showed no iron overload in either groups. There were also no differences in the parental report of vomiting (2 vs. 1), diarrhea (0 vs.0) or respiratory illness (6 vs. 3) between the NA and NNA group (all *p* > 0.05).

## Discussion

The present study provides an important update on the evidence regarding the effect of enteral iron supplementation on health outcomes in preterm infants with and without neonatal anemia. We found that daily iron supplementation reduced prevalence of ID/IDA during the first 6 months of life, with no iron overload and adverse effects on growth. Hb at birth and the history of blood transfusion were important factors associated with the prevalence of ID/IDA at 3 months. Sex, 3-month's Hb value and feeding patterns were important factors affecting ID/IDA at 6 months.

Without iron supplementation, preterm infants generally have an increased risk of developing ID/IDA. A Chinese and a Swedish cohort reported that the prevalence of anemia among preterm infants without iron supplementation was as high as 38.5 and 40.9%, respectively (10, 24). Consequently, specific iron supplementation is recommended in many studies. Some researchers recommend 2 mg/kg iron supplementation for preterm infants from 6 weeks to 6 months of age (10, 11), while others suggest that iron supplementation for the target population should be individualized (25). According to the consensus of experts in China, this study used a daily dose of 2 mg/kg of iron within 6 months. Through the follow-up, it was found that after 6 months' iron supplementation, the incidence of ID/IDA in NA and NNA group was quite similar. The total prevalence of IDA was only 15.8% at 6 months in our sample, while 20.8 and 54.3% in the general population in the same area of Zhejiang (23) and in southern China (26). These results indicated that the patient population in our study benefited from daily iron supplement before 6 months of age.

To further determine whether a history of neonatal iron supplementation and/or blood transfusion would influence the effectiveness of routine iron supplementation, we compared the iron status parameters in NAT and NAU group during neonatal hospitalization. Through analysis, we found that after daily iron supplementation, the incidence of ID/IDA in both groups was similar and no iron-overload was found. The results revealed that the daily dosage of 2 mg/kg external iron during early infancy was reasonable regardless of their neonatal treatment.

In our cohort, most of the preterm infants were maintained at an optimal iron status through iron supplementation during follow-up. However, ~15% of them still suffered from ID/IDA. As ID/IDA can have many adverse consequences, especially for the long-term detrimental effects on neurodevelopment, prevention, early detection and treatment are vitally important for the vulnerable population (27, 28). Therefore, we investigated factors affecting the occurrence of ID/IDA in the enrolled subjects at postnatal age of 3 and 6 months. We found that Hb at birth and the history of blood transfusion are two critical factors affecting the prevalence of ID/IDA during early postnatal life.

Infants with higher Hb level at birth had a decreased prevalence of ID/IDA, which indicated that iron status at 3 months of age depends largely on the iron storage at birth. It was consistent with the results from previous studies that iron endowment at birth were adequate to meet the requirements of premature infants for at least 2–3 months of life (29, 30). The larger the iron stores at birth, the greater the protection from the iron burden in early infancy (9, 31). Besides, we found that blood transfusion during neonatal hospitalization was another factor that reduced the risk of ID/IDA at 3 months. Red blood cell transfusion can increase hemoglobin serum levels and ferritin levels in preterm infants (32). Some researchers claimed that multiple RBC transfusions might tip the balance of iron homeostasis in premature infants and then lead to a higher risk of iron overload, and recommended caution in giving iron supplements in this patient population (33, 34). In our study, 4% infant had a history of multiple blood transfusions. After receiving daily iron supplementation during follow up, none of them developed iron overload. Due to the differences in age of blood testing and the sample size, we have not directly compare our current results with previous studies yet. But we speculated that human body has the ability to maintain iron homeostasis for a period of time after blood transfusion, for hepcidin may be an important factor involved in this process, acting as a negative feedback regulator for iron hemostasis after blood transfusion (32).

With age increasing, we found the above two factors were no longer major factors on the iron status. Instead, sex, Hb at 3 months and feeding patterns became the main factors at 6 months. In the present study, we found that male preterm infants had a 3.35-folder increased risk for developing ID/IDA compared to females. Similar findings were also reported in full-term infants, boys demonstrated lower Hb, MCV and ferritin, as well as higher ZPP and TfR than girls (35–37). Some studies attributed such male-female differences to growth-related factor and erythropoietic activity, as male infants grow faster and have higher erythropoietic activity than females, and thus have higher iron requirements (38). However, in our sample, under daily iron supplementation, the growth velocity was not an important factor affecting the iron status. Thus, the higher risk of ID/IDA cannot be simply attributed to growth-related factor, but is more likely to be related to other correlative factors. Domellöf et al. reported hormonal factors were responsible for the gender differences in iron metabolism (35). Nonetheless, our study has had no measures relevant to these intriguing possibilities.

In addition to sex, we found that Hb levels at 3 months also affected the prevalence of ID/IDA at 6 months. Around this time, iron from maternal storage and iron intake from milk becomes insufficient to meet the increasing needs for rapid catch-up growth and blood volume expansion, which leaves the preterm infants vulnerable to ID/IDA (34). Therefore, iron homeostasis at 6 months is more associated with the Hb levels at 3 months of age than Hb tested at birth and before discharge.

Similar to the prior studies in full-term infants (25, 37), we also found that preterm infants who were fortified fed, conventional formula-fed and mixed-fed had a lower risk of developing ID/IDA compared with infants who were exclusively breastfeeding, even in the case of daily iron supplementation. In the present study, none of the enrolled subjects had added complementary foods, so the total daily iron intake was calculated as the sum of iron supplements and iron intake from milk. Theoretically, the sum of all iron intake should be the same for preterm infants in different feeding patterns, with the dosage of 2 mg/kg per day. However, why is the feeding pattern still closely related to the iron status of preterm infants at the age of 6 months in our sample? We speculated that this may be attribute to better compliance with dairy milk intake than oral iron supplements. Further well-designed studies are needed to answer this question.

Some researchers have concerns about potential adverse effects of routine iron supplementation relate to iron overload and growth, especially in iron-sufficient infants (39). In our study, all the enrolled preterm infants, with or without a history of neonatal anemia, received daily iron supplementation starting at 40 weeks GA. We did find that routine iron supplementation was beneficial to maintain preterm infants' iron homeostasis, including those who were well-nourished or had a history of blood transfusion, with no iron overload observed. In addition, we also found that growth levels of infants in the NA group caught up with those in the NNA group at 3 and 6 months corrected age by controlling for BW, with rare side effects. The results above indicated that moderate iron supplementation was safe and did not have obvious adverse effects for preterm infants.

Taken together, these findings support our hypothesis that early iron supplementation is a critical strategy to prevent ID/IDA in preterm infants. A relative low iron intake at a dose of 2 mg/kg per day is appropriate for most premature infants in our follow-up cohort with and without neonatal anemia. Overall, few enrolled subjects had ID/IDA at 3 and 6 months, respectively. Preterm infants with higher level of Hb at birth and having a history of early treatment of blood transfusion may reduce the risk of developing of ID/IDA at 3 months; female infants, and those who had higher level Hb at 3 months or who were fed with formula or fortifier had a lower risk of ID/IDA at 6 months in the presence of daily iron supplementation.

Nevertheless, our study had several limitations. First, our study was limited by a relatively small sample size and short follow-up period, although comparable to some other studies. Second, the enrolled subjects were grouped based on the results of hemoglobin in their medical records, lacking iron status measures during neonatal period. Third, in the consideration of ethical issues, we did not set up a sub-group with normal iron status who were not provided with oral iron. Therefore, further well-designed longitudinal multicenter follow-up studies will be needed to expand the sample size of preterm infants with different iron status, to better monitor the long-term effect of routine iron supplementation among the target population and ultimately to determine if our results can be generalized.

## Conclusion

In summary, our study indicated that ID/IDA can be prevented effectively and safely with general iron supplementation at a dose of 2 mg/kg per day in preterm infants during the first 6 months of life. In the context of routine iron supplementation, Hb level at birth and early treatment of blood transfusion were two main factors affecting the iron status in early infancy; while Hb values at 3 months, sex and feeding patterns contribute to the iron status in the later stage among the patient population. Enteral iron supplementation in preterm infants should continue to be implemented, and iron status indicators such as hemoglobin, ferritin and ZPP/H should be regularly monitored during infancy.

## Data Availability Statement

The raw data supporting the conclusions of this article will be made available by the authors, without undue reservation.

## Ethics Statement

The studies involving human participants were reviewed and approved by the ethics committee of the Children's Hospital Zhejiang University School of Medicine (Permit Number: 2019-IRB-027). Written informed consent to participate in this study was provided by the participants' legal guardian/next of kin.

## Author Contributions

ML and CJ designed the research. ML, YL, JY, and LX conducted the research, carried out the statistical analyses, and interpreted the data. ML and YL drafted the initial manuscript and revised the manuscript. WC and QZ contributed to the laboratory testing, assisted with the analysis, and contributed to the revision of the manuscript. CJ and JS contributed to the critically revision of the manuscript. All authors approved the final content of the manuscript and agree to be accountable for all aspects of the work.

## Conflict of Interest

The authors declare that the research was conducted in the absence of any commercial or financial relationships that could be construed as a potential conflict of interest.

## References

[1] World Health Organization. The Global Prevalence of Anemia in 2011. (2021). Available online at: http://apps.who.int/iris/handle/10665/177094 (accessed March 27, 2021).

[2] Unger EL Hurst AR Georgieff MK Schallert T Rao R Connor JR. Behavior and monoamine deficits in prenatal and perinatal iron deficiency are not corrected by early postnatal moderate-iron or high-iron diets in rats. J Nutr. (2012) 142:2040–9. 10.3945/jn.112.162198 22990465 PMC3498975

[3] Santos DCC Angulo-Barroso RM Li M Bian Y Sturza J Richards B. Timing, duration, and severity of iron deficiency in early development and motor outcomes at 9 months. Eur J Clin Nutr. (2018) 72:332–41. 10.1038/s41430-017-0015-8 29235557 PMC5843498

[4] Lozoff B. Iron deficiency and child development. Food Nutr Bull. (2007) 28:S560–71. 10.1177/15648265070284S409 18297894

[5] Iglesias L Canals J. Effects of prenatal iron status on child neurodevelopment and behavior: a systematic review. Crit Rev Food Sci Nutr. (2018) 58:1604–14. 10.1080/10408398.2016.1274285 28084782

[6] McCarthy EK Dempsey EM Kiely ME. Iron supplementation in preterm and low-birth-weight infants: a systematic review of intervention studies. Nutr Rev. (2019) 77:865–77. 10.1093/nutrit/nuz051 31532494 PMC6888764

[7] Moreno-Fernandez J Ochoa JJ Latunde-Dada GO Diaz-Castro J. Iron deficiency and iron homeostasis in low birth weight preterm infants: a systematic review. Nutrients. (2019) 11:1090. 10.3390/nu11051090 31100900 PMC6566715

[8] Domellof M. Meeting the iron needs of low and very low birth weight infants. Ann Nutr Metab. (2017) 71(Suppl. 3):16–23. 10.1159/000480741 29268255

[9] Akkermans MD Uijterschout L Abbink M Vos P Rövekamp-Abels L Boersma B. Predictive factors of iron depletion in late preterm infants at the postnatal age of 6 weeks. Eur J Clin Nutr. (2016) 70:941–6. 10.1038/ejcn.2016.34 27004493

[10] Berglund S Westrup B Domellof M. Iron supplements reduce the risk of iron deficiency anemia in marginally low birth weight infants. Pediatrics. (2010) 126:e874–83. 10.1542/peds.2009-3624 20819898

[11] Berglund SK Westrup B Domellöf M. Iron supplementation until 6 months protects marginally low-birth-weight infants from iron deficiency during their first year of life. J Pediatr Gastroenterol Nutr. (2015) 60:390–5. 10.1097/MPG.0000000000000633 25406528

[12] Bora R Ramasamy S Brown B Wolfson J Rao R. Effect of iron supplementation from neonatal period on the iron status of 6-month-old infants at-risk for early iron deficiency: a randomized interventional trial. J Matern Fetal Neonatal Med. (2019) 2019:1–9. 10.1080/14767058.2019.1638358 31258019

[13] Angulo-Barroso RM Li M Santos DC Bian Y Sturza J Jiang Y. Iron supplementation in pregnancy or infancy and motor development: a randomized controlled trial. Pediatrics. (2016) 137:e20153547. 10.1542/peds.2015-3547 26936859 PMC4811316

[14] Zhang Y Zheng S Zhu L Ji C Angulo-Barroso RM Lozoff B. Impact of iron deficiency in ealry life stages on children's motor development: a longitudinal follow-up. Chin J Pediatr. (2019) 57:194–9. 10.3760/cma.j.issn.0578-1310.2019.03.007 30818896

[15] Franz AR Mihatsch WA Sander S Kron M Pohlandt F. Prospective randomized trial of early versus late enteral iron supplementation in infants with a birth weight of less than 1301 grams. Pediatrics. (2000) 106:700–6. 10.1542/peds.106.4.700 11015511

[16] Rao R Georgieff MK. Iron therapy for preterm infants. Clin Perinatol. (2009) 36:27–42. 10.1016/j.clp.2008.09.013 19161863 PMC2657918

[17] Perrone S Tataranno LM Stazzoni G Ramenghi L Buonocore G. Brain susceptibility to oxidative stress in the perinatal period. J Matern Fetal Neonatal Med. (2015) 28(Suppl. 1):2291–5. 10.3109/14767058.2013.796170 23968388

[18] Baker RD Greer FR. Diagnosis and prevention of iron deficiency and iron-deficiency anemia in infants and young children (0-3 years of age). Pediatrics. (2010) 126:1040–50. 10.1542/peds.2010-2576 20923825

[19] Wang D Liu X. Post-discharge feeding recommendations for premature, low birth weight infants. Chin J pediatr. (2016) 54:6–12. 10.3760/cma.j.issn.0578-1310.2016.01.003 27470474

[20] World Health Organization. Child Growth Standards: WHO Anthro (version 3.2.2, January 2011) and Macros. Geneva: WHO (2011).

[21] Cook JD Finch CA. Assessing iron status of a population. Am J Clin Nutr. (1979) 32:2115–9. 10.1093/ajcn/32.10.2115 484529

[22] World Health Organization. Haemoglobin Concentrations for the Diagnosis of Anaemia and Assessment of Severity. Vitamin and Mineral Nutrition Information System. Geneva: WHO (2011).

[23] Zhan J Zheng SS Dong WH Shao J. Predictive values of routine blood test results for iron deficiency in children. Chin J Pediatr. (2020) 58:201–5. 10.3760/cma.j.issn.0578-1310.2020.03.008 32135591

[24] Li Q Liang F Liang W Shi W Han Y. Prevalence of anemia and its associated risk factors among 6-months-old infants in Beijing. Front Pediatr. (2019) 7:286. 10.3389/fped.2019.00286 31355169 PMC6640653

[25] Yamada RT Leone CR. Hematological and iron content evolution in exclusively breastfed late-preterm newborns. Clinics. (2014) 69:792–8. 10.6061/clinics/2014(12)01 25627989 PMC4286666

[26] Luo R Shi Y Zhou H Yue A Zhang L Sylvia S. Anemia and feeding practices among infants in rural Shaanxi Province in China. Nutrients. (2014) 6:5975–91. 10.3390/nu6125975 25533008 PMC4277010

[27] Georgieff MK. Iron assessment to protect the developing brain. Am J Clin Nutr. (2017) 106:1588s−93. 10.3945/ajcn.117.155846 29070550 PMC5701704

[28] Andersson O Lindquist B Lindgren M Stjernqvist K Domellöf M Hellström-Westas L. Effect of delayed cord clamping on neurodevelopment at 4 years of age: a randomized clinical trial. JAMA Pediatr. (2015) 169:631–8. 10.1001/jamapediatrics.2015.0358 26010418

[29] Rao R Georgieff MK. Iron in fetal and neonatal nutrition. Semin Fetal Neonatal Med. (2007) 12:54–63. 10.1016/j.siny.2006.10.007 17157088 PMC2048487

[30] Saha B Jeeva Sankar M Gupta S Agarwal R Gupta N Deorari A. Iron stores in term and late preterm small for gestational age and appropriate for gestational age neonates at birth and in early infancy. Indian J Pediatr. (2016) 83:622–7. 10.1007/s12098-015-1960-7 26666906

[31] Ziegler EE Nelson SE Jeter JM. Iron stores of breastfed infants during the first year of life. Nutrients. (2014) 6:2023–34. 10.3390/nu6052023 24853888 PMC4042569

[32] Herzlich J Litmanovitz I Regev R Bauer S Sirota G Steiner Z. Iron homeostasis after blood transfusion in stable preterm infants - an observational study. J Perinat Med. (2016) 44:919–23. 10.1515/jpm-2015-0361 26992200

[33] Treviño-Báez JD Briones-Lara E Alamillo-Velázquez J Martínez-Moreno MI. Multiple red blood cell transfusions and iron overload in very low birthweight infants. Vox Sang. (2017) 112:453–8. 10.1111/vox.12528 28516443

[34] Amin SB Scholer L Srivastava M. Pre-discharge iron status and its determinants in premature infants. J Matern Fetal Neonatal Med. (2012) 25:2265–9. 10.3109/14767058.2012.685788 22734563

[35] Domellöf M Lönnerdal B Dewey KG Cohen RJ Rivera LL Hernell O. Sex differences in iron status during infancy. Pediatrics. (2002) 110:545–52. 10.1542/peds.110.3.545 12205258

[36] Choi JW Pai SH Im MW Kim SK. Change in transferrin receptor concentrations with age. Clin Chem. (1999) 45:1562–3. 10.1093/clinchem/45.9.1562 10471662

[37] Shao J Richards B Kaciroti N Zhu B Clark KM. Contribution of iron status at birth to infant iron status at 9 months: data from a prospective maternal-infant birth cohort in China. Eur J Clin Nutr. (2021) 75:364–72. 10.1038/s41430-020-00705-4 32814856 PMC7878278

[38] Choi JW Kim CS Pai SH. Erythropoietic activity and soluble transferrin receptor level in neonates and maternal blood. Acta Paediatr. (2000) 89:675–9. 10.1111/j.1651-2227.2000.tb00363.x 10914961

[39] Iannotti LL Tielsch JM Black MM Black RE. Iron supplementation in early childhood: health benefits and risks. Am J Clin Nutr. (2006) 84:1261–76. 10.1093/ajcn/84.6.1261 17158406 PMC3311916

